# Epigenetic Regulation of Cannabinoid-Mediated Attenuation of Inflammation and Its Impact on the Use of Cannabinoids to Treat Autoimmune Diseases

**DOI:** 10.3390/ijms22147302

**Published:** 2021-07-07

**Authors:** Bryan Latrell Holloman, Mitzi Nagarkatti, Prakash Nagarkatti

**Affiliations:** Department of Pathology, Microbiology and Immunology, University of South Carolina School of Medicine, Columbia, SC 29209, USA; bryan.holloman@uscmed.sc.edu (B.L.H.); mitzi.nagarkatti@uscmed.sc.edu (M.N.)

**Keywords:** chronic inflammation, autoimmune disease, endocannabinoids, phytocannabinoids, anti-inflammatory, epigenetics, Acute Respiratory Distress Syndrome

## Abstract

Chronic inflammation is considered to be a silent killer because it is the underlying cause of a wide range of clinical disorders, from cardiovascular to neurological diseases, and from cancer to obesity. In addition, there are over 80 different types of debilitating autoimmune diseases for which there are no cure. Currently, the drugs that are available to suppress chronic inflammation are either ineffective or overtly suppress the inflammation, thereby causing increased susceptibility to infections and cancer. Thus, the development of a new class of drugs that can suppress chronic inflammation is imperative. Cannabinoids are a group of compounds produced in the body (endocannabinoids) or found in cannabis (phytocannabinoids) that act through cannabinoid receptors and various other receptors expressed widely in the brain and immune system. In the last decade, cannabinoids have been well established experimentally to mediate anti-inflammatory properties. Research has shown that they suppress inflammation through multiple pathways, including apoptosis and inducing immunosuppressive T regulatory cells (Tregs) and myeloid-derived suppressor cells (MDSCs). Interestingly, cannabinoids also mediate epigenetic alterations in genes that regulate inflammation. In the current review, we highlight how the epigenetic modulations caused by cannabinoids lead to the suppression of inflammation and help identify novel pathways that can be used to target autoimmune diseases.

## 1. Introduction

Cannabis, often referred to as marijuana, is a flowering plant belonging to the family Cannabaceae. Traditionally, the cannabis inflorescence has been used for several centuries for both its medicinal and recreational properties. The flower contains over 500 distinct chemical entities, which include omega fatty acids, terpenoids, flavonoids, and phytocannabinoids. Currently, there are 120 known phytocannabinoids, which are oxygen-containing C21 aromatic hydrocarbon compounds found in marijuana [1,2,3,4]. While the chemical composition varies amongst cannabis species, the major bioactive and most abundant cannabinoids include Δ9-tetrahydrocannabinols (THC), tetrahydrocannabivarin (THCV), cannabidiol (CBD), cannabichromene (CBC), cannabinol (CBN), cannabidivarin (CBDV), cannabivarin (CBV), and cannabigerol (CBG). These cannabinoids are isolated at higher concentrations from cannabis feedstock compared to other cannabinoid compounds and are considered to be the key pharmacologic constituents of marijuana [5,6].

In recent decades, cannabinoids have received significant attention due to growing debate on their use for recreational and medicinal purposes. Consequently, advocacy for the medicinal benefits of phytocannabinoids such as THC remains overshadowed by its negative stigma for inducing psychoactive effects. Nevertheless, the therapeutic effects of THC are well documented, as evidenced by FDA approval of THC to alleviate vomiting and nausea stemming from chemotherapy, and treat cachexia in human immunodeficiency virus (HIV) patients, and the more recent use of a combination of THC and CBD from cannabis to treat spasticity in patients with multiple sclerosis (MS), which has been approved in many parts of the world [7,8,9]. The National Academy of Sciences released a report in 2017 in which they reviewed over 10,000 publications in the field of cannabinoids and concluded that while the use of cannabinoids against a wide range of clinical disorders remains to be established, cannabinoids are highly effective against chronic pain, chemotherapy-induced nausea and vomiting in cancer patients, and against an autoimmune disease, multiple sclerosis (MS) [10]. While there is strong experimental evidence to support the use of THC and other cannabinoids to treat various autoimmune disorders, the progress has been dampened by the regulatory barriers which make it difficult to conduct clinical trials, primarily due to the fact that THC is psychoactive and has been classified as a Schedule I drug [11]. Schedule I drugs are defined by the United States Drug Enforcement Administration as “drugs with no currently accepted medical use and a high potential for abuse”. This review aims to highlight the immunomodulatory properties of cannabinoids, which indicate their potential use in the treatment of inflammatory and autoimmune diseases.

There is significant experimental evidence demonstrating that cannabinoids exhibit potent anti-inflammatory properties. Cannabinoids have been well characterized for their anti-inflammatory properties, which they mediate through multiple pathways, including apoptosis [12,13,14,15], inhibition of cell proliferation [16], proinflammatory cytokine suppression [17,18], and T-cell polarization from proinflammatory to anti-inflammatory, such as from Th1 to Th2 and Tregs [19,20], as well as through the induction of highly immunosuppressive MDSCs [21]. In addition, cannabinoid receptor 2 (CB2) signaling suppresses the activation of proinflammatory (M1) microglia in neuroinflammation and shifts these cells into an alternative M2 activation state. M2 microglia are immunosuppressive cells [22,23]. Furthermore, endocannabinoid binding to astrocytes’ cannabinoid receptor 1 (CB1) exerts anti-tremor effects in an essential tremor animal model [24]. In contrast, some limited studies have also shown that cannabinoids can promote inflammatory cytokine production under certain conditions [25,26]. However, this review intends to focus on the practical application of therapeutic profiling using cannabinoid ligands, both agonists and antagonists, to target CB1, CB2, vanilloid receptors, and proliferator-activated receptor γ (PPAR-γ), which allows researchers and clinicians to exploit cannabinoids’ vast capabilities associated with treatment. In this review, we highlight recent findings on the role of marijuana cannabinoids as potential therapeutic agents against inflammatory and autoimmune disorders and specifically address the role of epigenetic pathways.

## 2. Role of CB1 and CB2 and Other Receptors in Inflammation

The phytocannabinoids, including the well-characterized THC and cannabidiol (CBD), are known to alter the activity of many receptors, including CB1 and CB2, 5HT1A, GPR55, μ- and δ-opioid receptors, TRPV1, and PPARγ [3]. Of these, the cannabinoid receptors are the most well characterized. CB1 receptors are predominantly expressed in presynaptic nerve cells in the central nervous system, including the limbic system, basal ganglia, hippocampus, and cerebellum. In contrast, CB2 receptors are commonly expressed in immune and gastrointestinal systems [27,28,29,30,31]. However, CB1 and CB2 receptors are occasionally expressed in lower densities outside of their traditional high-density habitats in other regions of the body. For example, CB1 receptors are expressed in the liver and thyroid gland [32] as well as in various immune cells [33], and CB2 receptors are expressed to some extent in the brain, primarily in microglial cells [34,35,36]. CB1 and CB2 differ in homology and reflect only a 44% protein level similarity, therefore allowing the different cannabinoid ligands to have a vast and unique range of selectivity and affinity for their receptors, resulting in multiple regulatory roles [27,37]. Activation of CB1 and CB2 receptors triggers high-affinity interactions with G protein with CB1 receptor activation leading to higher affinity interaction with G(i) and G(0), while CB2 activation leads to a high-affinity saturable interaction with G(i) but not efficiently with G(0) [38]. Interestingly, several structural classes of cannabinoid receptor ligands can induce a variety of signaling cascades due to their antagonistic behavior, partial vs. full agonistic capabilities, and their binding affinity, thus shedding light on the roles of receptor ligand efficacy. As such, stimulation of the subunits by different cannabinoids was shown to induce multifunctional mitogen-activated protein kinases, such as JUN-terminal kinase, phosphatidylinositol-3-kinase (PI3K,) and the p44/42 and p38 mitogen-activated protein kinases (MAPKs) pathway [12,39,40,41,42,43]. These metabolites activate a lipid-signaling system termed the endocannabinoid system, which is an essential biological regulatory system consisting of endocannabinods, receptors, and enzymes used in the biosynthesis and degradation of CB1 and 2 ligands [27,44].

Although CBD is not psychoactive, unlike THC, CBD is considered to be a negative allosteric modulator of the CB1 receptor, but the nearly identical structure, cannabidiol-dimethyl heptyl (CBD-DMH), is recognized as a mixed agonist/positive allosteric modulator [45]. Furthermore, CBD and CBD-DMH are both partial agonists of the CB2 receptor; however, CBD-DMH and CBD share separate binding sites of the CB2 receptor. While CBD displays a minimal effect on cAMP modulation, CBD-DMH acts as a positive allosteric modulator of cAMP modulation but serves as a negative allosteric modulator of β-arrestin1 recruitment; an intercellular regulatory protein that influences the analgesic side effects induced by some cannabinoid treatments [45,46]. While CBD’s relationship with the endocannabinoid system is well documented, CBD is also known to bind to the transient receptor potential vanilloid receptors (TRPVs), TRPV1 and TRPV2, inducing antihyperalgesic and anti-inflammatory effects [47,48,49], but THC shows no binding response to TRPV1. However, THC is a TRPV2, TRPV3, and TRPV4 agonist [3,49,50,51,52]. In addition, treatment with cannabidiol enhances peroxisome proliferator-activated receptor γ (PPARγ) transcription activity in MDSCs, leading to their mobilization [53]. THC also exerts agonistic effects after binding to PPARγ, stimulating vascular relaxation, and antitumor effects [54,55]. Unlike CBD, THC has strict structural and stereoselectivity specificities for CB1 receptors, which cause differences in drug–receptor interactions of CBD and THC [56,57]. For example, CBD has a lower affinity to the receptor than THC, with approximately 100 times less effective binding in the brain [58,59].

Interestingly, CBD has been shown to act as the opposite of THC and block the psychotropic properties of THC. Various mechanisms have been proposed to explain this, including antagonism at the CB1 receptor activation [60,61,62]. THC binding of the CB2 receptor induces multiple cellular responses. For example, THC stimulates proapoptotic behavior in human leukemia cells and cytotoxicity in J774-1 macrophages through activation of p38 MAPK [63,64]. Additionally, the binding of THC to the CB2 receptor induces immunomodulatory and immunosuppressive effects by decreasing cAMP production [36,65,66]. The wide array of receptors that the cannabinoids act on attests to various actions, signaling pathways that trigger and regulate several key physiological and immunological functions.

## 3. Cannabinoids and Mechanisms of Regulation of Inflammation

### 3.1. Cannabinoid-Induced Apoptosis

The detailed mechanisms through which cannabinoids induce apoptosis in immune cells, leading to suppression of inflammation, were reviewed in the past [67]. The phytocannabinoid-mediated proapoptotic response has been well documented in inflammatory states. Our lab and others have shown that THC induces apoptosis in a variety of immune cells, including T cells, B cells, macrophages, and splenic dendritic cells [13,14,15,68]. The activation of CB1 and CB2 receptors by THC triggers induction of caspases 2, 8, and 9 in bone marrow-derived DCs, leading to their apoptosis [67]. THC treatment was shown to induce apoptosis in antigen-presenting cells such as the DCs through phosphorylation of IkappaB-alpha and promote the transcription of several apoptotic genes controlled by NF-kappaB [15]. A pro-apoptotic response is also induced by CBD, leading to a reduction in proliferation and induction of apoptosis in cancer and immune cells [69,70,71]. CBD was shown to induce apoptosis in monocytes by acting on mitochondria to promote apoptosis through mitochondrial permeability transition pore opening and reactive oxygen species (ROS) production [72]. Using a select CB2 agonist, JWH-133, it was shown that this compound attenuated chronic colitis in IL-10 (−/−) mice as well as in mice with dextran sodium sulfate (DSS)-induced colitis [68]. In this model, JWH-133 induced apoptosis in activated T cells both in vivo and in vitro. These findings suggested that activation of CB2 leads to apoptosis in activated T cells, thereby reducing their numbers and attenuating colitis [68].

Similarly, using JWH-015, another synthetic CB2-selective agonist, it was shown that JWH-015 induced apoptosis in T and B cells that were activated with mitogens through induction of apoptosis [73]. JWH-015 was shown to enhance both the extrinsic and intrinsic pathways of apoptosis through activation of caspase-8, caspase-9, and caspase-3, as well as by inducing loss of mitochondrial membrane potential [73]. A recent study showed that THC attenuated SEB-mediated Acute Respiratory Distress Syndrome (ARDS) in mice by suppressing lung inflammation and blocking the cytokine storm. This effect was associated with immune cell apoptosis involving the mitochondrial pathway, as suggested by single-cell RNA sequencing [74].

### 3.2. Cannabinoids Induce Immunosuppressive MDSCs and Tregs

There are two main types of immunosuppressive cells: MDSCs and Tregs [19,21]. MDSCS are a heterogeneous group of myeloid cells that block the immune response by suppressing B cells’ and pro-inflammatory T cells’ proliferation, inhibit proinflammatory cytokine production, and induce apoptosis in activated cells [75,76]. In addition, MDSCs induce the formation of Tregs, and therefore may provide an immunosuppressive shield during an inflammatory state [77]. Regulatory T cells play an essential role in regulating self-tolerance and protect against autoimmunity by suppressing proinflammatory myeloid and lymphoid cells [78,79,80,81]. Interestingly, the immunosuppressive mechanistic responses of cannabinoids are linked to the induction of MDSCs and Tregs. One of the first studies which demonstrated that THC could induce, in mice, rapid and massive expansion of CD11b + Gr-1 + MDSCs came from our laboratory [82]. These cells expressed functional arginase, were highly immunosuppressive, and were induced following the production of granulocyte colony-stimulating factor (G-CSF) resulting from the activation of both CB1 and CB2 [82]. Subsequently, several other studies showed that cannabinoids, including CBD, can induce MDSCs [21,82].

Dhital et al. (2017) [19] reported that CBD increased Tregs, and Elliott et al. (2018) [21] reported that CBD induced MDSCs, which were responsible for the suppression of an experimental model of multiple sclerosis. CBD was also shown to activate PPAR-γ in mast cells, which trigger the induction of G-CSF, which in turn led to MDSC mobilization from the bone marrow [53]. Interestingly, endocannabinoids have also been shown to trigger MDSCs. For example, 2-AG, an endogenous cannabinoid, was shown to attenuate neuroinflammation by increasing the number and functions of MDSCs in the brain [83]. Additionally, administration of AEA was also found to induce MDSCs, which consisted of both granulocytic and monocytic subtypes [84]. These cells were shown to be immunosuppressive by the fact that adoptive transfer of MDSCs attenuated mBSA-induced delayed hypersensitivity reaction. In this study, AEA was found to induce MDSCs through activation of CB1 receptors [84].

While cannabinoid ligands are accompanied by their ability to activate anti-inflammatory immune cells, the polarization of T-cell differentiation from Th1 to Th2 T helper cells is also notable. THC increases mRNA expression of Th2 cytokines while decreasing the expression of mRNA coding for Th1 cytokines, and shifts the balance of Th1–Th2 through histone modification [20,85]. Using JTE907, a selective/inverse agonist of CB2, it was shown this compound promoted the differentiation of Th0 cells into Tregs by inducing FoxP3, TGF-β, and IL-10 [86]. In this study, the authors used DNBS-induced colitis and showed that JTE907 treatment induced CD4 + CD25 + FoxP3 + cells in the lamina propria, which was associated with attenuation of colitis [86]. Using low levels of T-cell stimulation, CBD was shown to enhance the differentiation of CD25+FOXP3+ cells from CD4+, CD4+ CD25+, and CD4+CD25− T cells, and such cells were found to be highly immunosuppressive [19]. Additionally, AEA upregulated CD200 and IL-10 but downregulated pro-inflammatory cytokines IL-1β and IL-6 in a mixed culture of neurons and reactive microglia activated by LPS and IFNγ [87]. The CD200–CD200R axis enhanced by AEA is believed to shift microglial cells from an M1 to M2 phenotype, which provides neuroprotection [88]. Furthermore, CB1 agonist WIN55,212-2 induces astrocytes to release purines, which reduce tremors in an animal model of essential tremors [24].

## 4. Epigenetics Pathways of Cannabinoid-Mediated Suppression of Inflammation

**Figure 1:**
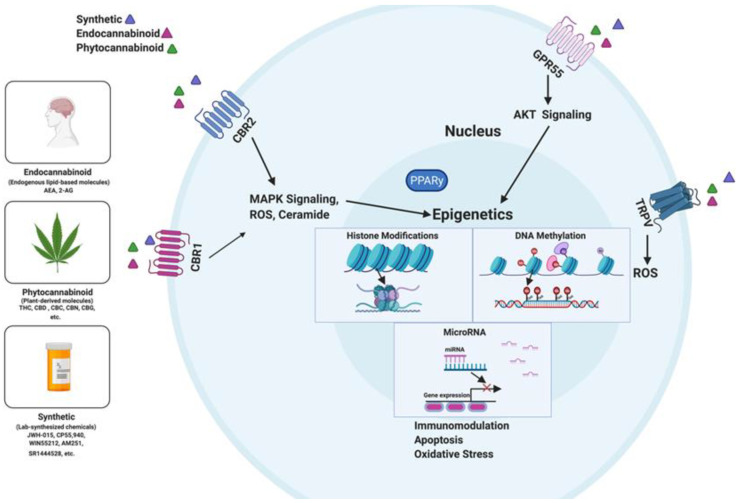
An overview of cannabinoid-induced epigenetic alterations through DNA methylation, histone modifications, and miRNA dysregulation. Several classes of cannabinoids, which include synthetic cannabinoids, endocannabinoids, and phytocannabinoids, regulate inflammation by binding to several receptors (CB1 and CB2, GPR55, and TRPV) found on immune cells. Activation of the CB1 and 2 leads to MAPK signaling transduction and increases the production of ROS and ceramide. The MAPK pathway induces epigenomic changes. On the other hand, GPR55 activation leads to AKT signaling transduction, which stimulates epigenetic alterations.

Epigenetic alterations play a critical role in gene expression and can occur because of environmental factors, including diet-derived constituents [89]. These are heritable alterations that can impact gene function and are not dependent on the DNA sequence. Recent studies have suggested that epigenetic pathways can also regulate inflammation [90]. Cannabinoids have been shown to promote epigenetic changes through DNA methylation and acetylation, histone modification, and alterations in the expression of microRNAs (miRNAs) [91]. Fingerprinting the epigenome in response to cannabinoids provides insights into how gene expression involving immune cell differentiation and function may be altered by cannabinoid exposure or treatment. Interestingly, the mechanisms of anti-inflammatory effects mediated by cannabinoids can be linked in most instances to epigenetic modulations. We have tried to highlight the studies on epigenetic regulation of inflammation by cannabinoids below (Figure 1).

### 4.1. Role of miRNA in Cannabinoid-Mediated Suppression of Inflammation

In vivo, THC-treated mice downregulate the expression of miR-17/92 and miR-374b/421 clusters and increase expression of miR-146a following exposure to Staphylococcal Enterotoxin B (SEB), a bacterial superantigen that activates a large number of T cells and induces cytokine storm and Acute Respiratory Distress Syndrome (ARDS). It was interesting to note that exposure to SEB triggered 100% mortality in mice, and it was shown that THC treatment led to 100% survival of such mice, which was associated with the miRNA changes in immune cells [92]. In this study, SEB, which induced the miRNA-17-92 cluster, specifically miRNA-18a, was shown to target Pten (phosphatase and tensin homologue), which inhibits the PI3K/Akt signaling pathway, thereby preventing the generation of Tregs, while THC reversed this action of the miRNAs, thereby promoting the generation of Tregs. miR-146a upregulation correlates with an increase in cellular apoptosis and a decreased expression of proinflammatory cytokines interleukin (IL)-6 and -12 and interferon (IFN)γ [93,94]. In combination, THC and CBD were shown to suppress neuroinflammation in an experimental model of multiple sclerosis through downregulation of miR-21a-5p, miR-31-5p, miR-122-5p, miR-146a-5p, miR-150-5p, miR-155-5p, and miR-27b-5p, and upregulation of miR-706-5p and miR-7116. The downregulated miRs targeted molecules involved in cycle arrest and apoptosis, such as CDKN2A, BCL2L11, and CCNG1, thereby inducing them to promote apoptosis, as well as SOCS1 and FoxP3, which are involved in suppressing inflammation [17].

Cannabinoids, including THC and CBD, have been shown to induce highly immunosuppressive MDSCs [75,77]. MDSCs inhibit B cells’ and pro-inflammatory T cells’ proliferation, suppress proinflammatory cytokine production, and induce apoptosis in activated cells [75,76]. Induction of MDSCs may result from epigenetic changes. For example, THC was found to robustly induce miRNA-690 in THC-derived MDSCs, which targeted transcription factor CCAAT/enhancer-binding protein α (C/EBPα) involved in the differentiation of MDSCs [95].

THC has also been shown to suppress delayed-type hypersensitivity (DTH) reaction [96]. THC treatment suppressed inflammatory Th17 cells in this DTH model by downregulating miR-21, which decreased Th17 differentiation via SMAD7 induction. Additionally, THC increased the expression of miR-29b, thereby inhibiting IFN-γ and thus, the Th1 response [96]. It is interesting to note that endogenous cannabinoids such as anandamide have also been shown to suppress the DTH response mediated by Th17 and Th1 cells through downregulation of cytokines such as IL-17 and IFN-γ, and increase the anti-inflammatory cytokine IL-10 [97]. Anandamide was found to profoundly impact miRNA expression in immune cells and induced several miRNAs that targeted the inflammatory cytokines [97].

SIV-infected rhesus macaques develop intestinal inflammation, and when THC was administered into such animals, it attenuated SIV disease progression with reduced viral replication and inflammation [98]. Interestingly, a number of microRNAs that targeted inflammatory markers were shown in this study to be upregulated, thereby suppressing inflammation, including miRs-10a, -24, -99b, -145, -149, and -187. Of these, miR-99b specifically targeted NOX4, thereby decreasing this protein which is known to cause damage to intestinal epithelial cells through oxidative stress [98].

### 4.2. Role of DNA Methylation in Cannabinoid-Mediated Suppression of Inflammation

DNA methylation leads to covalent modification of DNA with methyl groups in the promoter region of genes at the CpG islands. This is carried out by DNA methyltransferases and usually leads to decreased transcription. As discussed above, THC was shown to induce immunosuppressive MDSCs and in this context, THC was shown to increase methylation in MDSCs at the promoter region of DNMT3a and DNMT3b and caused a decrease in promoter region methylation of Arg1 and STAT3, key molecules involved in the differentiation and functions of MDSCs [96]. Interestingly, in THC-treated SIV-infected rhesus macaques, it was found that approximately half of the differentially expressed genes had altered DNA methylation [99]. In this study, some of the hypermethylated genes were involved in inflammation, such as C/EBPD, which controls the expression of IL-1, IL-6, and TNF-α.

### 4.3. Cannabinoid-Mediated Histone Modifications and Their Impact on Inflammation

Post-translational modification of core histone proteins has been shown to impact chromatin structure, which leads to either activation or repression of the related genes. For example, histone modifications involving H3K4me3 and H3K36me3 were shown to activate the associated genes, while those involving H3K9me3 and H3K27me3 cause gene repression. Some examples of histone modifications include methylation, acetylation, phosphorylation, and ubiquitination.

While THC has been shown to alter T-cell differentiation from Th1 to Th2, whether this is related to histone modifications was explored in mice exposed to SEB [85]. In this model, THC induced suppressive histone marks in the Th1-associated genes and activating histone marks in Th2 genes [85]. Another study showed that anandamide protected neurons from inflammatory damage, which was associated with upregulation of mitogen-activated protein kinase phosphatase-1 (MKP-1) in microglial cells with histone H3 phosphorylation of the mkp-1 gene [100]. This led to attenuation of MAPK signal transduction in microglial cells and dampened the inflammation. Long noncoding RNAs (lncRNAs) have been shown to play a critical role in gene expression, from histone modification to stability of proteins. Recent studies from our lab showed that an lncRNA called AW112010 was significantly induced in activated CD4+ T cells and CBD or THC could decrease the expression of AW112010 in T cells [101]. Additional studies showed that lncRNA AW112010 suppressed IL-10, thereby promoting the differentiation of inflammatory T cells through histone demethylation [101]. Thus, cannabinoids could induce immunosuppressive cytokines such as IL-10 through downregulation of lncRNA AW112010.

## 5. Use of Cannabinoids to Treat Multiple Sclerosis (MS)

Multiple sclerosis (MS) is a chronic autoimmune disease in which inflammation in the central nervous system (CNS) leads to demyelination and paralysis. It was shown that myelin antigen-specific Th1 and Th17 T cells are primarily responsible for causing the destruction of myelin sheath in the CNS [102]. While the etiology is not well understood in humans, myelin oligodendrocyte glycoprotein and proteolipid protein peptides (MOG and PLP, respectively) induce experimental autoimmune encephalomyelitis (EAE) in mice, a model used extensively to study MS. These antigens drive Th1 and Th17 cellular proliferation and migration to the CNS and cause a cytokine inflammatory storm that consists of IL-1B, IL-2, IL-17-, and IFNγ, damaging myelin sheath [17,103,104,105,106,107,108].

Interestingly, cannabinoids and CB1 and CB2 receptors have been shown to play a critical role in the regulation of EAE and MS. For example, activation of CB1 and CB2 receptors leads to attenuation of neuroinflammation and EAE [82,109], while blocking CB1 using SR141716A, an antagonist, accelerated the clinical onset and development of EAE [110]. Cannabinoids have been shown to attenuate EAE-associated spasticity, in which a 20% peak reduction in spasticity signs was reported in mice treated with a low-dose of Sativex (5 mg/kg THC + 5 mg/kg CBD), which has been approved for clinical use in many countries. However, a high dosage of Sativex (10 mg/kg THC + 10 mg/kg CBD) causes a 40% reduction in spasticity in treated mice [111]. The fact that the combination of THC+CBD (10 mg/kg each) can reduce not only spasticity, but also suppress neuroinflammation caused by Th17 and Th1 cells, was shown in a recent study in which it was demonstrated that these cannabinoids decreased inflammatory TNF-α-secreting CD4 T cells, and TBX21 expression. Furthermore, THC+CBD treatment increased anti-inflammatory cytokines and transcription factors, including IL-10, IL-4, TGF-β, FoxP3, and STAT5b, which are involved in the attenuation of EAE [17,112]. The combination of cannabinoids also triggered apoptosis in immune cells found in the brain, which was associated with downregulation of miR-21a-5p, miR-31-5p, miR-122-5p, miR-146a-5p, miR-150-5p, miR-155-5p, and miR-27b-5p while upregulating miR-706-5p and miR-7116 [112]. Furthermore, the majority of the miRs that were decreased targeted molecules that regulated cycle arrest and apoptosis, such as CDKN2A, BCL2L11, and CCNG1, and anti-inflammatory molecules such as SOCS1 and FoxP3 [112].

In addition to the use of a combination of THC+CBD, CBD alone has also been tested for its efficacy against EAE, and it was shown to delay EAE disease onset and clinical signs in C57BL/6 mice [21,113,114]. In one report, the inflammatory cytokines, IFNγ and IL-17, were elevated in the sera of EAE mice while treatment with CBD reduced these cytokines [21]. Transcription factors, retinoic acid receptor-related orphan receptor gamma (RORγT) and T-bet expression were decreased in splenic T cells after treatment with CBD as well [21]. Further, the expression of IL-10 increased after CBD treatment, revealing an immunosuppressive phenotype induced by CBD [21]. In vitro studies using CBD revealed similarities to in vivo data inasmuch as splenocytes cultured from EAE-CBD mice, when stimulated with MOG35-55 peptide for three days, showed a decrease in IFNγ, TNF-α, and IL-17 when compared to cells from EAE-VEH mice. Furthermore, cannabinoid receptor type 2 ligand (−)-B-caryophyllene (BCP), a terpene and phytocannabinoid, has similar capabilities as CBD, and exhibited anti-inflammatory and analgesic effects in murine EAE models through inhibition of TNF-α and IL-1B [18]. BCP binds selectively to the CB2 receptor and acts as an agonist, inhibiting proinflammatory chemokines and restoring catalase along with superoxide dismutase and glutathione peroxidase activities [18,115,116,117,118].

While the effect of CBD-treatment on cytokine production and transcription factor activity is well documented, CBD also has been shown to induce the infiltration of immunosuppressive myeloid-derived suppressor cells (MDSCs) in vivo [21]. Administration of CBD intraperitoneally into mice facilitated an influx of CD11b+Gr-1+ MDSCs into the peritoneal cavity. However, while treatment with CBD failed to increase the infiltration of CD11b+Gr-1+ MDSCs into the CNS of EAE mice, MDSCs isolated from intraperitoneal lavage were able to suppress EAE when transferred into naïve mice, thereby demonstrating conclusively that MDSCs induced by CBD can suppress neuroinflammation [21].

miRNA has been shown to play a significant role in the regulation of MS or EAE. For example, miR223 deficiency leads to attenuation of CNS inflammation, and demyelination during EAE by increasing autophagy in brain microglial cells [119]. Studies from our laboratory showed that miRNA let-7e was induced in EAE, and that let-7e silencing in vivo inhibited the induction of Th1 and Th17 cells and thereby ameliorated EAE. In this study, let-7e was found to target IL-10, an immunosuppressive cytokine [120]. In EAE, cannabinoids’ regulatory role in the suppression of inflammation may be through alterations in the expression of several miRNAs by activating cannabinoid receptors [121]. Specifically, THC+CBD treatment downregulated the expression of several miRNAs such as miRNA31, miRNA 21a, miRNA 146a, miRNA 155, and miRNA 33. It is noteworthy that SOCS1, CCNG1, FoxP3, Bcl2L11, IL-10, and FoxP3 are involved in immunosuppression and/or apoptosis and are direct targets of these miRNAs. In addition, CBD treatment in MOG-stimulated cells enriches H3K4me3 in the FoxA1-binding motif [122]. FoxA1+ Treg cells’ immunosuppressive properties are associated with the attenuation of EAE symptoms [123].

Furthermore, the miR-7116-5p, which targets genes encoding IL-6 and TNF-α, is upregulated after THC+CBD treatment. These cytokines are essential for EAE progression [21]. In summary, cannabinoids including CBD and THC have been shown to be effective in the treatment of EAE or MS and several studies have shown that some of the anti-inflammatory activities may be mediated through miRNA.

### 5.1. Inflammatory Bowel Disease (IBD)

IBD is used to describe two clinical disorders that are triggered by chronic inflammation in the gastrointestinal (GI) tract: ulcerative colitis (UC) and Crohn’s disease (CD). CD can affect any part of the GI tract, primarily the portion of the small intestine before the start of the large intestine or colon, while UC primarily impacts the colon and rectum. Alterations in the mucosal microenvironment and prolonged activation of proinflammatory immune cells in the intestines destroy the inner lining, leading to an increase in intestinal epithelial permeability and ulcers [124,125,126,127]. As shown by our lab and by many others, T cells play an essential role in ulcerative colitis onset, outlining the destructive functionality of CD4+ T helper cells subsets, Th1 and Th17 [128]. The severity of IBD has been linked to Th1 production of IFNγ and TNF-α, and Th17 secretion of IL-17A [129,130,131,132,133]. However, the influx of immunosuppressive cells in the intestinal microenvironment through the use of phytocannabinoids is quickly gaining attention for treating IBD. Thus, cannabinoids and the endocannabinoid system may mediate protection against intestinal inflammation and help in the healing of colitis by activating immunosuppressive myeloid and lymphoid cells.

Cannabis is commonly used by many patients suffering from IBD. While there is some clinical data to suggest cannabis use helps attenuate IBD symptoms and improve the quality of life, there are no reports on increased clinical remission from IBD [134]. However, there are a large number of studies using animal models, which have shown that cannabinoids are highly effective in attenuating colitis. Interestingly, THC, when used alone or in combination with CBD treatment, suppresses inflammation and ameliorates 2,4,6,-trinitrobenzene sulphonic acid (TNBS)-induced acute colitis in rodents [135]. In DSS-induced colitis, THC or THC+CBD treatments reduce polyps in colon cancer-induced mice and increase CD103+ dendritic cells and CD4+Nrp1+FoxP3+ T regulatory cells (nTregs) in the lamina propria through activation of the CB2 receptor [136]. The increase in the ratio of CD4+Nrp1+FoxP3+ cells in the lamina propria reduces disease severity in THC- and THC+CBD-treated colitis mice compared to VEH-treated colitis mice [136]. However, the increase in barrier integrity and mucus production following treatment with THC or THC+CBD were linked to the CB1 receptor [136]. Like THC, CBD alone has also been reported to attenuate TNBS-induced colitis [137]. Pretreatment with CBD reduced the expression of the cannabinoid ligand-receptor GPR55 and lowered myeloperoxidase levels (MPO) and IL-6 [137]. However, TNF-α levels were not significantly altered in CBD-TNBS mice vs. VEH-TNBS mice. In addition, changes in colonic motility were noted following treatment with CBD [137]. For example, CBD increased downward colonic motility but slowed upper motility, and this may be due to CBD pretreatment preventing Ca2+ -ATPase activity changes in smooth muscle cells [137].

While T cells have been noted in the pathology of IBD and colitis severity, macrophages also play a pivotal role in colitis and may serve as another avenue for managing and treating intestinal inflammatory diseases. Romano et al. (2013) reported that cannabichromene (CBC) ameliorates DNBS-induced colitis by reducing nitric oxide, IFNγ, and IL-10 levels in colitis-associated macrophages [6]. In addition, other immunosuppressive cells, such as MDSC, may help ameliorate colitis in mice following phytocannabinoid treatment [53].

Another interesting biologic property of phytocannabinoids is their ability to alter intestinal microRNA expression. THC induces an anti-inflammatory microenvironment in the intestine by upregulating miR-10a, miR-24, miR-99b, miR-145, miR-149, and miR-187 expression; miRNAs with proinflammatory targets [98]. Interestingly, miR-10a targeted dendritic cell-associated proinflammatory cytokines IL-12/IL-23p40 and assisted with maintaining gut homeostasis [138], while miR-187 increased IL-10 production and downregulated TNF-α production in LPS-activated monocytes [139]. miR-99b mediated downregulation of NOX4, a reactive oxygen species generator gene, has been shown to protect the intestinal epithelium from oxidative stress-induced damage [98]. Moreover, THC treatment in uninfected macaques upregulated miR-141 expression in the duodenum and decreased the expression of migrating inflammatory cells by downregulating the expression of CXCL12 in the intestinal lamina propria [98]. As discussed earlier, in simian immunodeficiency virus (SIV)-infected macaques, treatment with THC suppressed viral replication and intestinal inflammation and delayed the disease progression [98]. This effect was associated with increased expression of miR-10a, miR-24, miR-99b, miR-145, miR-149, and miR-187, which are known to target proinflammatory signaling pathways. Recent studies from our laboratory demonstrated that THC-mediated activation of CB2 prevented colitis-associated colon cancer [140]. THC increased the expression of CD103 on macrophages and DCs and increased the expression of TGF-β1, which caused an increase in Tregs, which contributed towards suppressing colitis and colon cancer [136].

### 5.2. Autoimmune Hepatitis

Autoimmune hepatitis (AIH) is a chronic inflammatory liver disease characterized by an autoantigen-mediated immune response leading to cirrhosis and resulting in liver failure [141]. The criteria for AIH diagnosis is based on histologic features, autoantibodies, serum immunoglobulin G levels, elevated alanine aminotransferase (ALT) and aspartate transaminase (AST), negative viral serology, inflammation, and infiltrating immune cells [142,143,144]. The activation of hepatic stellate cells (HSCs) and liver fibrosis progression is a major cellular event of hepatitis [145,146]. Interestingly, the endocannabinoid system involvement in the progression and regression of liver disease has been reported by multiple labs, and novel therapeutic strategies using cannabinoids have been proposed to analyze its capabilities in reversing autoimmune hepatitis to replace medications, such as prednisone and azathioprine, due to their long-term side effects.

It is known that cannabinoid receptors are expressed in the liver and hepatic immune cells during disease onset, and activation of these receptors affects the disease outcome [147,148,149]. In cirrhotic livers, human HSCs express CB1, and increased expression has been linked to liver fibrogenesis [147,148,149]. The ablation of CB1 using pharmaceuticals or gene therapy protects from liver injury, and with the ablation of CB1 using medicines or gene therapy, mice are protected from liver injury [147,148,149]. While this suggests that chronic liver injury may be mediated by activation of the endocannabinoid system [150], there is also experimental evidence to suggest that cannabinoids may be highly effective in treating certain forms of immune cell-mediated liver injury [151]. This discrepancy may arise from the diverse etiological agents that trigger liver injuries, the nature of the immunological model used to trigger hepatic injury, and the different immune mechanisms that cause this liver injury.

The current review focuses on immunological models of liver injury, such as autoimmune hepatitis (AIH), in which cannabinoids have been shown to have beneficial effects. Concanavalin A (ConA)-induced hepatitis in mice is a well-established model for human AIH. Injection of ConA, a polyclonal activator of T cells, into mice, triggers cytokine storm, infiltration of immune cells in the liver, and development of hepatitis. In this model, administration of 20 or 50 mg/kg THC suppressed proinflammatory cytokines, increased anti-inflammatory forkhead helix transcription factor p3+ (FoxP3) T regulatory cells, and decreased liver enzymes such as AST and ALT, thereby protecting the liver from immune assault [151]. THC also suppressed proinflammatory cytokines such as TNF-α, IFN-γ, and IL-6, and significantly increased CD4+CD25+Foxp3+ regulatory T cells [151]. It is noteworthy that CB1/CB2 agonists CP55,940 and WIN55212 had similar effects as THC [151]. Additionally, pretreatment with CB1 antagonist AM251 or CB2 antagonist SR1444528 in ConA+THC-injected mice prevented THC-mediated effects in ConA-induced hepatitis, thereby showing that THC acted through both these receptors [151]. The endogenous cannabinoid, anandamide, has also been shown to attenuate AIH in mice by suppressing cytokine levels. In addition, deficiency or inhibition of fatty acid amide hydrolase (FAAH), an endocannabinoid hydrolyzing enzyme, leads to increased anandamide levels, which attenuates AIH induced by ConA [151]. In mice, THC, in an ultra-low dose (THC: 0.002 mg/kg), attenuates hepatic oxidative stress through the CREB pathway and reduces elevated ALT and AST levels [152]. THC has also been shown to attenuate apoptosis in hepatic cells, alleviating hepatic ischemia/reperfusion (I/R) injury [152]. Together, such studies demonstrate that cannabinoid receptor agonists can be used to treat AIH.

Moreover, our lab has also shown that treatment with CBD ameliorates experimental AIH by attenuating acute inflammation in the liver [153]. Treating mice with CBD after ConA challenge reversed hepatitis tissue liver injury by decreasing liver enzyme levels and proinflammatory cytokine levels and increasing CD11b+Gr-1+MDSCs, highly immunosuppressive cells of myeloid linage [153]. In addition, proinflammatory cytokines TNF-α, IFNγ, and MCP-1, and eotaxin-1 (CCL11), IL-2, IL-6, IL-12(p-40), and IL-17 were significantly decreased in ConA+CBD-treated mice compared to ConA+Vehicle-treated mice. Interestingly, CBD failed to induce MDSCs and ameliorate liver injury in vanilloid receptor-deficient mice (TRPV1−/−), signifying that CBD acted through the TRPV1 vanilloid receptor to induce liver MDSCs and liver injury suppression [153].

Based on studies published from our laboratory on the efficacy of CBD to treat experimental AIH, the FDA has granted orphan drug status, a special status given to a drug to treat a rare illness, for CBD to treat human AIH. It is worth noting that in a recent survey involving human AIH, in those who were using CBD, the majority of the responders reported a significant improvement in pain (82%), sleep (87%), and fatigue (61%) [154]. Additionally, some of these CBD users indicated that they were able to stop a prescription medication because of the efficacy of CBD [155].

## 6. Conclusions

The complexity of the endocannabinoid system, including cannabinoid receptors, metabolizing enzymes, and exogenous cannabinoids, allows for its multifunctional role in suppressing inflammation by acting through several pathways. The mechanism through which phytocannabinoids attenuate inflammatory and autoimmune diseases includes induction of apoptosis in activated immune cells, T-cell polarization from pro-inflammation to anti-inflammation, and induction of immunosuppressive cells such as Tregs and MDSCs. Interestingly, some of these mechanisms are also regulated by the epigenetic alterations triggered by cannabinoids. Thus, epigenetic pathways induced by cannabinoids may play a critical role in the regulation of inflammation by these compounds both endogenously and exogenously. Identification of epigenetic pathways induced by cannabinoids that suppress inflammation also help identify novel pathways that can be directly targeted to prevent or treat inflammatory and autoimmune diseases.

While there is clear experimental evidence on the efficacy of cannabinoids to treat inflammatory and autoimmune diseases, clinical trials are lacking because some cannabinoids such as THC are psychoactive and classified under Schedule 1 drugs. Nonetheless, more recently, THC+CBD (Sativex) is being used in many parts of the world to treat spasticity in MS, and the FDA has approved CBD as an orphan drug to treat autoimmune hepatitis.

Studies have shown that while CB2 receptor activation can lead to suppression of inflammation, use of CB1 receptor-based therapeutics, such as agonists and antagonists, can cause neuropsychiatric adverse effects. Thus, CB2 agonists that are non-psychoactive may serve as an effective therapy for inflammatory and autoimmune diseases. However, CB2 agonists and THC can also increase pro-inflammatory cytokines under certain conditions [156,157]. Clearly, clinical trials are necessary to study the efficacy of CB2 receptor-based therapies.

CBD has also been shown to exert anti-inflammatory properties, although its efficacy may be slightly less than THC. However, due to the non-psychoactive property of CBD, it may be better suited to treat human inflammatory and autoimmune diseases. The fact that it is readily available currently as a dietary supplement may discourage clinical trials being pursued. Additionally, the fact that endocannabinoids can also regulate inflammation suggests that manipulating their endogenous levels by blocking enzymes such as FAAH may also serve as a novel therapeutic agent to treat inflammation; however, using an FAAH inhibitor, such as BIA 10-2474, in high dosage can be highly toxic [158]. Clearly, additional studies are necessary to explore such possibilities.

In summary, there is strong experimental evidence to suggest that endogenous and exogenous cannabinoids regulate inflammation and thus provide unique tools for developing novel drugs that target the cannabinoid receptors or endocannabinoids for attenuating inflammation. This, combined with clinical trials using cannabinoids found in cannabis, may help explore their efficacy in the treatment of autoimmune disease.
